# Diagnosis and surgical approach of adult Hirschsprung's disease: About two observations and review of the literature. Case series

**DOI:** 10.1016/j.amsu.2019.10.017

**Published:** 2019-10-25

**Authors:** Harissou Adamou, Ibrahim Amadou Magagi, Oumarou Habou, Ousseini Adakal, Maman Bachir Aboulaye, Alliance Robnodji, Lassey James Didier, Rachid Sani, Habibou Abarchi

**Affiliations:** a General and Digestive Surgery - Zinder National Hospital, Faculty of Health Sciences, University of Zinder, Niger; b Pediatric Surgery - Zinder National Hospital, Faculty of Health Sciences, University of Zinder, Niger; c General and Digestive Surgery - Faculty of Health Sciences, Dan Dicko Dan Koulodo University of Maradi, Niger; d General and Digestive Surgery, National Hospital of Niamey, Faculty of Health Sciences, Abdou Moumouni University of Niamey, Niger; e Pediatric Surgery, National Lamordé Hospital, Faculty of Health Sciences, Abdou Moumouni University of Niamey, Niger

**Keywords:** Hirschsprung's disease, Bowel obstruction, Transanal endorectal pull-through procedure, Delayed coloanal anastomosis, Adult, Case series

## Abstract

**Introduction:**

Hirschsprung's disease (HD) is uncommon in adulthood. In this study, we describe the management of two cases of adult Hirschsprung's disease treated with transanal colonic pull-through procedure followed by a delayed coloanal anastomosis.

**Patients and methods:**

This was a retrospective (December 2016 to Jun 2019) study included two cases of adult HD with confirmed Hirschsprung disease who underwent surgery at Zinder National hospital, Niger. The registration number is researchregistry 5174.

**Results:**

These were two patients aged 21 years (male) and 22 years (female) admitted to the emergency department with an acute bowel obstruction. The history finds a delayed passage of meconium at birth with a history of long-standing recurrent constipation since early childhood for the 2 patients. A lateral colostomy was performed urgently in both patients and the barium enema revealed a disparity of the sigmoid colon with corn shaped transition zone. Histologic examination of the biopsy specimen confirmed the diagnosis of HD. Surgery was done according to transanal endorectal pull-through procedure followed by delayed coloanal anastomosis. Patients were regularly followed over a period of 16 months. Constipation was gone, no continence problem was reported and quality of life was rated satisfactory.

**Conclusion:**

The discovery of Hirschsprung's disease is rare in adulthood. Transanal endorectal pull-through procedure followed by delayed coloanal anastomosis with conventional surgery is a suitable option for the treatment of HD and gives a good result.

## Highligths

• More than 90% of cases, diagnosis and treatment of Hirschsprung’s disease are made in the first 5 years of life; as a result, the diagnosis of HD in adulthood is rare.
• Surgical treatment in adulthood is more laborious because of anatomical changes.
• Many surgical procedures have been described, the best known of which are Swenson, Duhamel, Soave and Lynn. Modifications of these techniques have been proposed.
• The goal of surgical treatment is to resect or exclude the aganglionic segment and to lower the healthy colon, normally innervated at the anus.
• Transanal colonic pull-through procedure followed by a delayed coloanal anastomosis with conventional surgery and manual anastomosis, gives satisfactory results and remains well adapted to the adult Hirschsprung’s disease in context of limited resources.

## 1 Introduction

Hirschsprung's disease (HD) is a congenital malformation of the distal end of the gastrointestinal tract characterized by an absence of neuronal ganglion cells of the Meissner (submucosal) and Auerbach (muscular) nerve plexuses over a segment of variable length [[1], [2], [3], [4]]. It is a rectosigmoid lesion in 80% of cases [4]. This rare disease (1/5000 live births) results to a permanent spasm of the aganglionic segment and leads to progressive dilation of the upstream colon [1,2,4]. It is a condition traditionally diagnosed and treated in infancy, but diagnosis of HD in adulthood is rare [2,3]. Diagnosis of HD is delayed in some settings by ignorance of the patient, lack of knowledge and habitual long-term treatment of chronic constipation [2,3,5]. Stubborn constipation is the main circumstance of discovery, more rarely an acute intestinal obstruction [2,3,5,6]. Numerous surgical procedures have been described, the best known of which are Swenson, Duhamel, Soave and Lynn [2,[7], [8], [9], [10]]. The goal of surgical treatment is to resect or exclude the aganglionic segment and to lower the healthy colon, normally innervated at the anus while preserving the sphincter function [1,2]. Modifications of these techniques have been proposed in a minimally invasive approach [2,3,11,12]. Through this study, we report our experience in the management of two cases of adult Hirschsprung's disease treated by the rectosigmoid resection with a transanal colonic pull-through procedure followed by a delayed coloanal anastomosis.

## 2 Patients and methods

This work has been reported in a line the PROCESS criteria [13]. This article had been registered in accordance with the declaration of Helsinki at the Research 2019. The registration number is Researchregistry 5174. The ethical approval was obtained from a joint decision of the Scientific Council of the Faculty of Health Sciences of Zinder University and the Advisory Technical Board of Zinder National Hospital, Ref: FSS-UZ/HNZ-CTC-0023-02-03-2017.

This was a retrospective study included two cases of adult Hirschsprung's disease with histologic confirmation, who were operated at Zinder National Hospital (public), Niger, West Africa. Both patients were admitted to the hospital with mechanical bowel obstruction. Data were collected from the clinical and surgical records between 11th December 2016 and 4th Jun 2019. We recorded for each patient: age, sex, history, clinical and results of radiological examination (barium enema), histologic result, standard preoperative investigations, and the follow up after surgery.

Excluded from the study were adult patients with suspicion of Hirschsprung's unconfirmed histology or patients who had not been operated on.

### 2.1 Surgical procedure

**Fig. 1:**
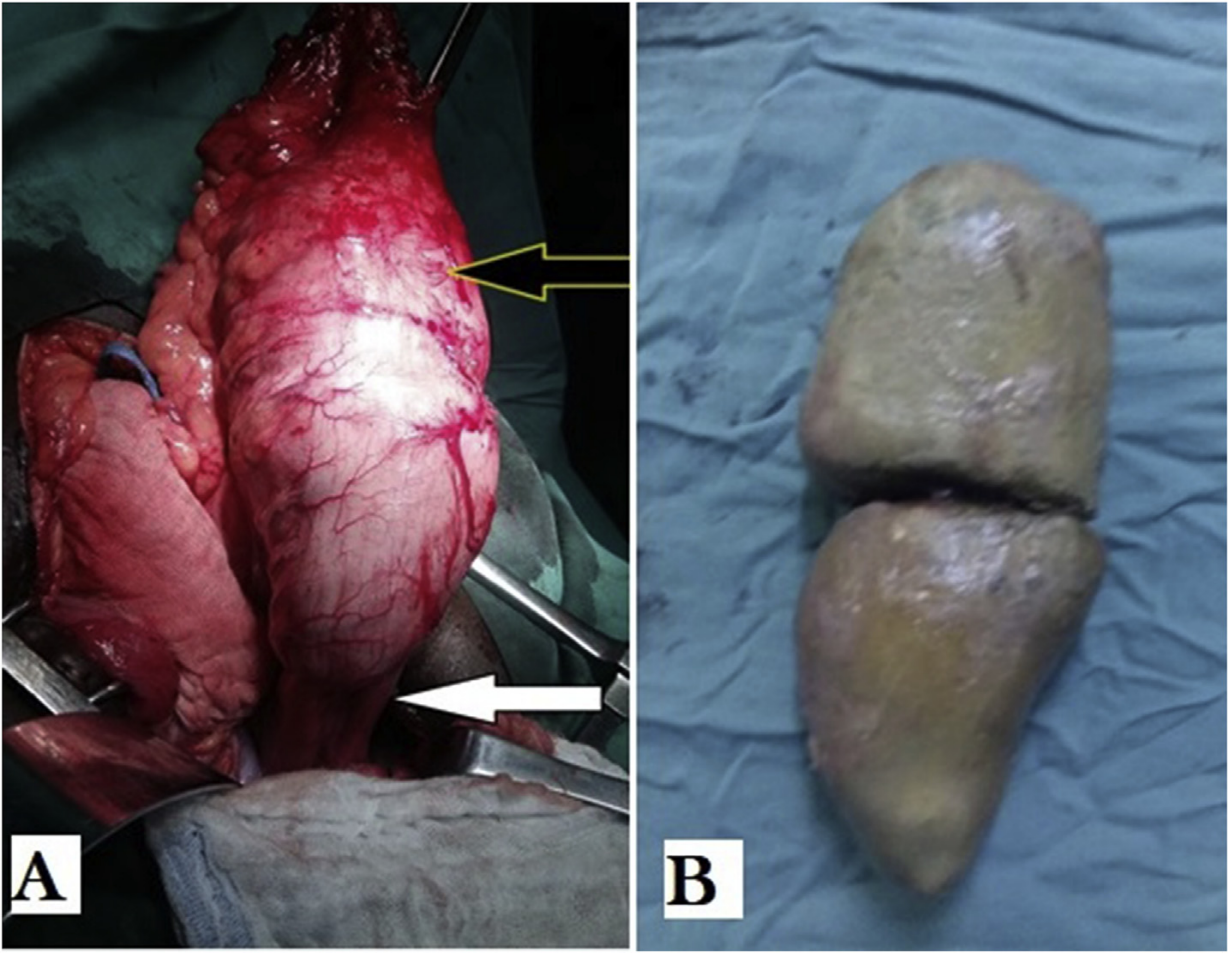
A-Intraoperative view showing the size disparity between the narrowed area (white arrow) and the dilated area (yellow arrow), B- Aspect of fecal extracted in the recto-sigmoid. (For interpretation of the references to color in this figure legend, the reader is referred to the Web version of this article.)

The colostomy was performed at emergency until definitive surgical treatment. Left colon and rectum were markedly dilated and impacted stool was extracted intraoperatively (Fig. 1).

**Fig. 2:**
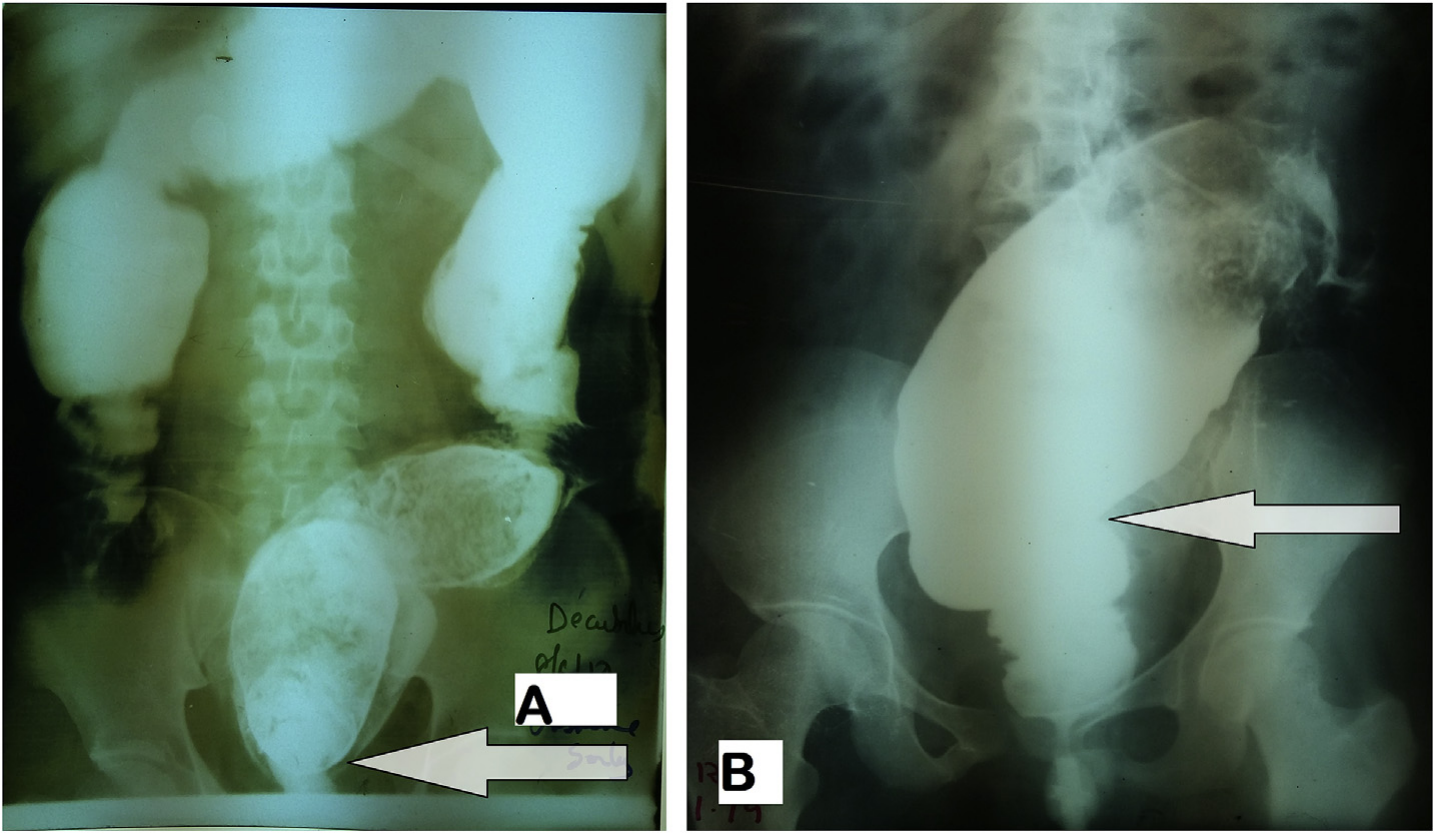
Barium enema showing a distended A- at the lowest rectum in male patient with a narrowed rectal area (arrow). B- colon with narrowed area (Arrow) in the middle 1/3 of the rectum in female patient.

A barium enema (Fig. 2) and preoperative biological assessment (blood count, uremia, creatinine, blood glucose, hemostasis) were done after colostomy. The Left iliac colostomy was retained for a period of 3 months for both patients. Bowel preparation for several days was made prior to surgery in order to reduce fecal overload. Surgery was performed under general anesthesia by the surgery was done by a team of senior surgeons and junior residents. We performed a modified Soave's procedure inspired by Faucheron and Jarry's approach [2,3] that consisted of a laparoscopic rectosigmoid resection with a transanal colonic passage procedure followed by a delayed coloanal anastomosis without protective stoma. Surgical procedure was done in two stages. The patients were placed in a double approach: abdominal and perineal.

**Fig. 3:**
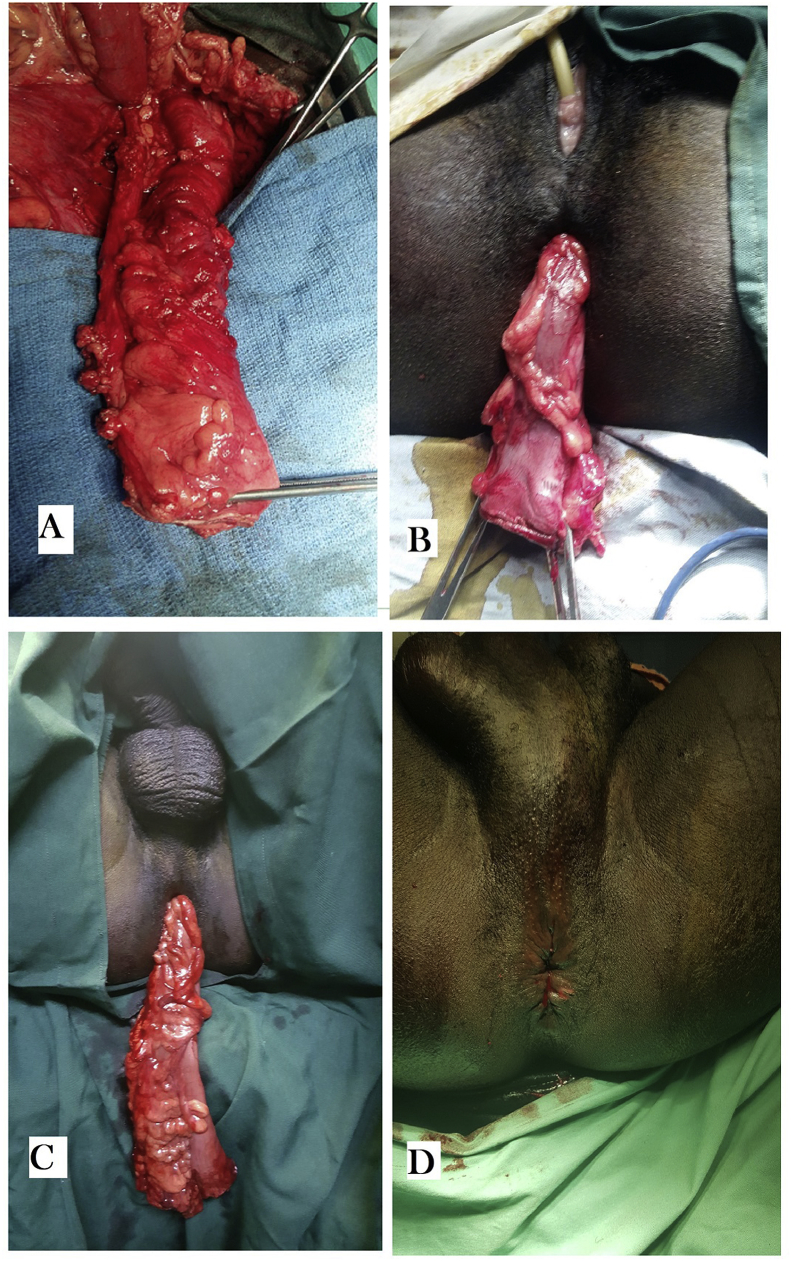
Intraoperative view A-colon released beyond the pubic symphysis. B-colon lowered transanal in female. C- Transanal lowered colon in male. D-Aspect of the anus after the coloanal anastomosis.

The first stage of our procedure consisted to rectosigmoid resection by laparotomy followed by transanal colonic pull-through by perineal approach and delayed coloanal anastomosis. The patient is placed in a Lloyd-Davies position. At laparotomy, the colostomy is taken down. The colonic angle was released sigmoid arteries and veins were linked. The sluggish part of the sigmoid colon and rectum was resected. The contractile colon under the flap was released and its distal end protruded from the symphysis rim by 12–25 cm (Fig. 3). The rectal stump was dissected posteriorly until the pelvic floor at the upper edge of the external sphincter with preservation of the mesorectum. At the perineal phase, the patients were placed in a lithotomy position with Trendelenburg. Anal retractor was used in order to dilate the anal canal. To reduce bleeding and facilitate dissection adrenaline xylocaine (2%) was infiltrated into the submucosa of the lower rectum above the dentate line. A mucosectomy of the posterior hemi-circumference of the rectum was made from the dentate line to the upper edge of the external sphincter (identified by the index placed in a posterior hook). Then the dissection is carried at right angles to cross the muscularis and join the level of the abdominal dissection by realizing a wide rectal gap. A long Babcock clamp is passed upward through the rectal opening. The sigmoid retrorectal, transrectal and then transanal lowering of the colon is achieved and the colon is externalized through the anus. The lowered colon exceeded the anal margin by at least 10 cm without tension (Fig. 3). The rectal stump therefore remains in front of the lowered colon. The serosa of the colon is in direct contact with the rectal muscularis. The abdominal cavity is closed on a drain. The externalized colon was fixed by 2 stitches at the anal margin and tied to the inside of the right thigh. The distal tip was opened to allow the elimination of gas and stool.

The second surgical stage was scheduled under general anesthesia 7 days after the first stage in the lithotomy position. The resection of the excess of the colon was done as well as the manual coloanal anastomosis. The patients were observed for 30 days postoperatively and follow-up was carried out over a period of at least 12 months.

## 3 Results

During the study period, 2 patients underwent rectosigmoid resection by laparotomy with a transanal colonic pull-through followed by delayed manual coloanal anastomosis.

Both patients were classified as ASA 1. The history finds a notion of delayed passage of meconium at birth, chronic constipation and episodes of repeated subocclusion. These patients were living in an undeveloped rural area and had never visited a modern health center for his symptomatology. They use traditional treatments to fight constipation and often defecated with very hard stools by performing digital maneuvers, until the day they were admitted in surgical emergency department with clinical condition of acute intestinal obstruction. The abdomen was very distended with diffuse tympanism for each patient. Demographics data, clinical and radiological characteristics of patients are summarized in Table 1.

**Table 1:** Demographic, clinical and radiological characteristics of patients.

Characteristics	Patient 1	Patient 2
Age at diagnosis (years)	22	21
Sex	Female	Male
Marital Status	Married	Unmarried
Profession	Housewife	Farmer
Patient's locality	Rural area (370 km from Zinder)	Rural area (550 km from Zinder)
History of symptoms	At birth	At birth
Body mass index (BMI) Kg/m^2^	20.2	22.17
Blood pressure (mmHg)	100/66	120/70
General status	Good	Good
Digital rectal examination	Empty rectal ampoule	Fecal impaction
Transition zone at barium enema	Narrow Rectosigmoid	Narrow lower rectum
Standard Laboratory investigation	Normal	Normal
Peroperative view at emergency	Volvulus of sigmoid	colonic distention
Lenght of Hospital stay (days)	15	17
Follow up (months)	16	18

At laparotomy, there was a huge distention of the whole colon with a twisting of the sigmoid colon counterclockwise in one case. After devolvulation, a colostomy according to Bouilly-Volkman procedure was performed, and fecal impaction was extracted intraoperatively (Fig. 1). The diagnosis Hirschsprung's disease was mentioned. The barium enema was required at 1 month after the colostomy shows a huge colorectal dilation with a transition zone narrowed to the 1/3 middle of the rectum for one case (Fig. 2-A), and a narrowed area of the lower rectum for the other case (Fig. 2-B). There was also significant stercoral stasis upstream of the transition zone.

The full-thickness surgical rectal biopsy performed in the narrowed area confirmed an absence of ganglion cells. The diagnosis of short form Hirschsprung's disease was retained based on the patient history, clinical data, radiological findings and histological examination of the full-thickness rectal biopsy showing the absence of ganglion cells in the nerve plexuses. Surgery was performed three months after colostomy for both patients. A rectosigmoid resection by laparotomy with a transanal colonic pull-through was done followed by delayed manual coloanal anastomosis.

### 3.1 Postoperative

Both patients tolerated the colostomy well until definitive surgical treatment. The mean of length stay at the hospital was 16 days. Postoperative follow-up within 30 days was uneventful for both patients.

### 3.2 Follow-up

**Fig. 4:**
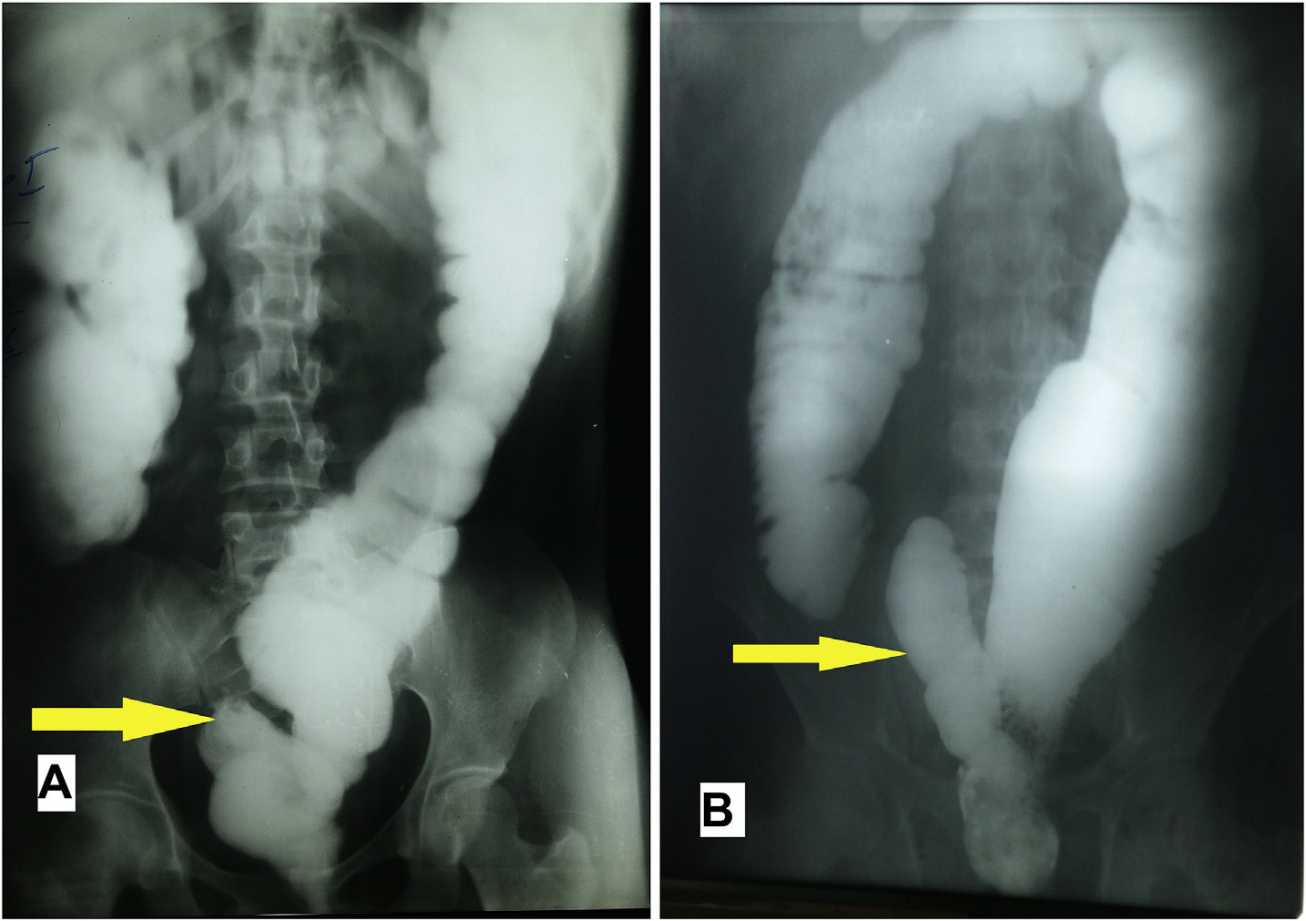
Barium enema after surgery showing the rectal stump left in place (arrow). A -Female, B- Male.

The two patients all testified 12 months after leaving the hospital that their quality of life improved significantly. The male patient was followed up for a mean period of 17 months Patients had no longer stubborn constipation or abdominal pain. Radiological control by a barium enema was done at 3 months of the intervention showed good passage without stercoral stasis (Fig. 4).

## 4 Discussion

This study reports only 2 cases of adult HD and to our knowledge these are the 1st case reported in Niger Republic. Conventional surgery was used in our study. We carried out rectosigmoid resection by laparotomy with transanal colonic pull-through. The lowered colon was fixed temporarily and the preventive diverting stoma was not necessary since the intestinal transit was preserved. Manual coloanal anastomosis was delayed until 7 days after the first surgical phase. This is well reproducible and the results obtained in management these two patients are satisfactory. This is Soave's procedure modified by Faucheron and Jarry [2,3] in a minimally invasive approach; which consisted of a laparoscopic rectosigmoid resection with transanal colonic pull-through and delayed coloanal anastomosis.

Harald Hirschsprung is the first who published the classic description of HD in 1887 [14]. This is a neurocristopathy because the ganglion cells of the neurenteric system are derived from the neural crest [[1], [2], [3]]. HD is one of the leading causes of intestinal obstruction in children. In more than 90% of cases, diagnosis and treatment are made at an early age in the first 5 years of life; as a result, the diagnosis of HD in adulthood is rare [[1], [2], [3], [4], [5], [6],[15], [16], [17]]. For some authors, the expression « adult Hirschsprung's disease » is used when the diagnosis is made after ten years old [4]. Moreover, Parc et al. [18] said: « *there is no Hirschsprung's disease that is revealed in adulthood, there is only HD unknown until adulthood*». The first case of HD in adults was reported by Rosin et al. [19] in 1951. Doodnath et al. [4] in a meta-analysis reported 490 cases of HD in adults between 1950 and 2009. Since the number of HD in adults has greatly exceeded 600 cases in the literature [5,[15], [16], [17]]. In our context of inadequate perinatal and pediatric care, the diagnosis of HD in adulthood is due to a lack of awareness of the symptoms, on the one hand, and difficulties in accessing quality care on the other hand [5]. Our patients were 21 years old and 22 years old. According to the literature, the diagnosis of the adult HD is often made between 20 years and 40 years old [[4], [5], [6],[15], [16], [17]]. In our patients, HD was diagnosed during an acute intestinal obstruction due to fecal impaction. In the majority of cases, the diagnosis is evoked during the interview, which specifies a persistent constipation since childhood or sometimes the parents report the notion of delayed passage of meconium at birth reported by the parents [[2], [3], [4], [5], [6],[15], [16], [17]]; like in our study. In some cases there is chronic abdominal pain such as colic, loss of weight [2,3]. The classic clinical features of HD in adults are abdominal distention, rectal emptiness or, on the contrary, the presence of a hard fecal impaction detect by rectal examination [2,17]. Sometimes the rectal examination, by lifting the rectal spasm, reveals a "glove finger" sensation in the lower forms of HD [3]. Radiological examinations for diagnostic purposes are barium enema, and computed tomography which highlights colonic dilation with a narrowed area corresponding to the aganglionic zone [2,3]. Anorectal manometry shows a sphincterial hypertonia and especially an absence of the recto-anal inhibitory reflex [2,16]. This exploration is not available in our context. As in children, the formal confirmation of the diagnosis of HD is based on the histologic examination of the operative specimen or a colorectal panparietal biopsy [2,3,17]. Histologic examination of the biopsy specimen confirmed the diagnosis of HD for our patients. Transanal colonic pull-through procedure followed by a delayed coloanal anastomosis was used to operate our two patients.

The surgical technique of adult HD should take into account anatomical changes and the difficulty of dissection in adulthood [2,15]. There are many surgical procedures described in the treatment of HD, but 4 main techniques dominate: Swenson, Duhamel, Soave and Lynn procedures [2,[7], [8], [9], [10],^12^]. The first technique was described by Swenson [7] in 1948 consists of a sigmoidorectal resection by removing the aganglionic part and the distended upstream colonic part with coloanal anastomosis after eversion of the rectal stump through the anus.

When the coloanal anastomosis is performed at the same time, a stoma of protection is needed to reduce the risk of anastomosis leakage and sepsis [2]. An ileostomy or an upstream colostomy is performed in principle [2,7]. In this study, we received our patients in emergency with an acute intestinal obstruction. The first procedure of treatment at emergency consisted of left iliac colostomy. After a period of 3 months, our patients were prepared and operated on a double abdominal and perineal approach using modified Soave's procedure inspired by Faucheron and Jarry [2,3,8]. This technique is well adapted to our surgical practice with limited resources. When the colon is lowered about 10 cm, an ostomy is not necessary and the manual coloanal anastomosis is performed a few days later [2]. Some techniques such as De La Tore and Ortega-Salgado's procedure [12] which consisted in making the surgical treatment of HD in children by exclusive perineal approach. This operation is not suitable for adults, but offers the advantages of a minimally invasive approach, a shorter hospitalization, early recovery of complete diet, less pain and improved aesthetics with good functional results [12]. For some authors, the surgical treatment of HD in adults may benefit from the advantages of a minimally invasive approach by performing a sigmoidorectal resection by laparoscopy, followed by a transanal lowering of the healthy colon without stoma. Indeed, the fact of externalizing the colon by the anal route allows to do without a stoma until the coloanal anastomosis is made [[1], [2], [3],^11^]. This approach gives a better aesthetic and functional result than the classical techniques described by Duhamel, Swenson and Soave [2]. Transanal endorectal pull-through procedure (TERPT) followed by delayed coloanal anastomosis leaving a rectal stump in the place was easily performed by our team. This technique is still current for somes authors and gives satisfactory results in the treatment of HD [2,3,11]. According to Yao et al. [11], the benefits of the Duhamel and TERPT procedures are similar to the treatment of Hirschsprung's disease. However, there are differences in the length of postoperative hospital stay and the incidence of enterocolitis [11]. Duhamel's procedure is the most practiced technique worldwide [4,17]. According to a systematic review of 490 cases of HD after childhood, 47.2% of Duhamel's intervention was performed, 10% of Swenson's procedure, 8.2% of Soave's technique and 9.2% of myomectomy alone [4]. However, in the series of Ducan et al. [15], the Soave and Swenson procedures were used respectively 55.5% and 45.5% of cases, leading to anastomotic stenosis in one patient treated with the Soave procedure and fistula in two patients treated with the Swenson procedure. In our study, after a follow-up of more than 16 months, patients do not show any functional signs related to HD or the treatment. However, in this study, as in most adult HD series, hindsight is not sufficient to study long-term quality of life [1,3,20]. There are few studies evaluating the long-term outcomes of surgical treatment of adult HD [4,20]. The follow-up period is short (16 and 18 months) in our study. However patients have a good clinical course and no longer complain of constipation or abdominal pain. The two patients all testified after leaving the hospital that their quality of life improved significantly.

Limitations: This study reported only the surgical management of two cases of HD in adults. The technique initially used by Jarry and Faucheron was mini-invasive. We performed an invasive technique by laparotomy and manual anastomosis. This procedure used, does not require a preventive stoma, and shows anastomotic security. But this small number does not allow us to make an exhaustive comparison with the data of the literature.

## 5 Conclusion

Adult Hirschsprung's disease is rare. The diagnosis is often referred to a major and persistent constipation evolving since birth. In some even rarer situations, the diagnosis is evoked during a laparotomy for acute mechanical bowel obstruction. The treatment is surgical and several procedures have been described. Colorectal resection by laparotomy with transanal colonic pull-through and delayed coloanal anastomosis is a practical and valid option for the treatment of Hirschsprung's disease in adults. We believe that this procedure gives satisfactory results and remains well adapted to the adult HD. It is a good alternative in context of limited resources that do not have a technical platform for the minimally invasive approach. Using this procedure (TERPT followed by delayed coloanal anastomosis) with a larger series and longer follow-up could help us build more on long-term functional results.

## Ethical approval

The ethical approval was obtained from a joint decision of the Scientific Council of the Faculty of Health Sciences of Zinder University and the Advisory Technical Board of Zinder National Hospital, Ref: FSS-UZ/HNZ-CTC-0023-02-03-2017.

## Sources of funding

No funding was received for this research.

## Author contribution

Harissou Adamou, Ibrahim Amadou Magagi, Oumarou Habou: have conceived and designed the study, written and drafted the manuscript. Ousseini Adakal, Maman Bachir Aboulaye, Alliance Robnodji, Lassey James Didier, Rachid Sani, Habibou Abarchi: have all contributed to the management, writing. All authors in this manuscript contributed to drafting and writing of this manuscript and approved the final manuscript.

## Trial registry number

1. Name of the registry: Research Registry.
2. Unique Identifying number or registration ID: Researchregistry 5174
3. Hyperlink to the registration (must be publicly accessible): https://www.researchregistry.com/browse-the-registry#home/

## Guarantor

Harissou Adamou.

## Consent

Written informed consent was obtained from the patient for publication of these case report and accompanying images. A copy of the written consent is available for review by the Editor-in-Chief of this journal on request.

## Provenance and peer review

Not commissioned, externally peer reviewed.

## Declaration of competing interest

None.
